# Surface Functionalised Optical Fibre for Detection of Hydrogen Sulphide

**DOI:** 10.3390/bios13110949

**Published:** 2023-10-24

**Authors:** Shaghayegh Baghapour, Jasmine Nehema, Wen Qi Zhang, Stephen C. Warren-Smith, Shane M. Hickey, Sally E. Plush, Shahraam Afshar Vahid

**Affiliations:** 1 Laser Physics and Photonic Devices Laboratory, UniSA STEM, University of South Australia, Adelaide, SA 5095, Australia; wenqi.zhang@unisa.edu.au (W.Q.Z.); stephen.warren-smith@unisa.edu.au (S.C.W.-S.); shahraam.afsharvahid@unisa.edu.au (S.A.V.); 2 Clinical and Health Sciences, University of South Australia, Adelaide, SA 5000, Australia; jasmine.nehema@mymail.unisa.edu.au (J.N.); shane.hickey@unisa.edu.au (S.M.H.); sally.plush@unisa.edu.au (S.E.P.); 3 Future Industries Institute, University of South Australia, Adelaide, SA 5095, Australia

**Keywords:** hydrosulphide ion detection, 1,8-naphthalimide, turn-on fluorescent probes, optical fibre, fluorescence intensity enhancement, X-ray photoelectron spectroscopy

## Abstract

Dysregulated production of hydrogen sulphide in the human body has been associated with various diseases including cancer, underlining the importance of accurate detection of this molecule. Here, we report the detection of hydrogen sulphide using fluorescence-emission enhancement of two 1,8-naphthalimide fluorescent probes with an azide moiety in position 4. One probe, serving as a control, featured a methoxyethyl moiety through the imide to evaluate its effectiveness for hydrogen sulphide detection, while the other probe was modified with (3-aminopropyl)triethoxysilane (APTES) to enable direct covalent attachment to an optical fibre tip. We coated the optical fibre tip relatively homogeneously with the APTES-azide fluorophore, as confirmed via x-ray photoelectron spectroscopy (XPS). The absorption and fluorescence responses of the control fluorophore free in PBS were analysed using UV-Vis and fluorescence spectrophotometry, while the fluorescence emission of the APTES-azide fluorophore-coated optical fibres was examined using a simple, low-cost optical fibre-based setup. Both fluorescent probes exhibited a significant increase (more than double the initial value) in fluorescence emission upon the addition of HS^−^ when excited with 405 nm. However, the fluorescence enhancement of the coated optical fibres demonstrated a much faster response time of 2 min (time for the fluorescence intensity to reach 90% of its maximum value) compared to the control fluorophore in solution (30 min). Additionally, the temporal evolution of fluorescence intensity of the fluorophore coated on the optical fibre was studied at two pH values (7.4 and 6.4), demonstrating a reasonable overlap and confirming the compound pH insensitivity within this range. The promising results from this study indicate the potential for developing an optical fibre-based sensing system for HS^−^ detection using the synthesised fluorophore, which could have significant applications in health monitoring and disease detection.

## 1. Introduction

Hydrogen sulphide (H_2_S) is a crucial signaling gasotransmitter molecule endogenously generated in the human body [1,2,3,4,5,6]. While normal concentrations have beneficial health effects, abnormal production of H_2_S is associated with a range of prevalent diseases including diabetes, Alzheimer’s, hypertension and liver cirrhosis [2,4,5,7,8]. Additionally, it has been reported that H_2_S is involved in cancer [9,10]. Cancer cells overexpress the enzymes which produce H_2_S, resulting in the elevated levels in malignant tissues [9,11,12]. An increased concentration of H_2_S can lead to tumour growth, proliferation, angiogenesis, invasion, metastasis and chemotherapy resistance [9,12,13]. Therefore, accurate detection and monitoring of H_2_S in living systems holds significant importance.

Conventional methods such as gas chromatography [14] and chemical sensing based on semiconductive metal-oxide [15] and electrochemical analysis [16] have been commonly used to detect H_2_S. Despite their advantages, the use of these techniques to detect H_2_S in living systems is limited due to their poor stability and flexibility, complicated sample preparation and inability to perform real-time monitoring [4,5,17,18,19]. Fluorescence-based methods to detect H_2_S are attracting increasing research interest due to their high sensitivity and selectivity [20,21,22]. One strategy which has been employed within biological settings is to exploit the ability of H_2_S to reduce non-fluorescent aromatic azides to fluorescent aromatic amines [2,3,6,23,24]. For example, when an azide functional group is incorporated onto a 1,8-naphthalimide backbone, the molecule exhibits weak fluorescence emission due to the strong electron-withdrawing nature of the azide group. Upon the addition of hydrogen sulphide, the reduction of the azide group to an electron-donating amino group results in an intramolecular charge-transfer process, which leads to an increase in emission intensity [25]. In physiological conditions, it is the conjugate base HS^−^ that is primarily present, as H_2_S has a pKa near 7; therefore, while there are reports of H_2_S in solution, it is the more nucleophilic HS^−^ anion rather than the diprotic H_2_S that is being sensed. Further, Henthorn and Pluth [26] have shown it is the HS^−^ anion that is responsible for aryl azide reduction. Several research works have been conducted to successfully analyse endogenous hydrogen sulphide in living cells and tissues using azide-functionalised 1,8-naphthalimide probes [4,20,25]. However, these methods have been limited by the need to add exogenous probes to the cellular environment, which could potentially increase the risk of cytotoxicity or effect cellular processes themselves. Moreover, they exhibit constraints in regard to response time [4,20,27].

Recently, optical fibres have gained significant attention in the development of compact, remote and real-time sensing platforms due to their flexibility, light weight, small size and immunity to electromagnetic interference [28,29]. Furthermore, optical fibres can be functionalised using various techniques for sensing purposes, thereby eliminating the need for adding exogenous agents to the biological samples. Researchers typically employ different configurations and mechanisms including surface plasmon resonance and localised surface plasmon [1,30,31,32], fibre gratings [33,34] and photonic crystal fibres [35,36] to detect gaseous H_2_S. However, it should be noted that these methods capable of detecting H_2_S gas may not be directly applicable in biological contexts in which the target molecule for detection is HS^−^ in solution rather than H_2_S. A significant category of fibre-optic sensors relies on fluorescence and has emerged as a successful method for detecting various molecules and biomolecules [37,38,39,40,41,42]. Fluorescence-based fibre optic detection of non-fluorescent analytes can be achieved through the immobilization of fluorescent probes on optical fibre surface, either by immobilisation of probes via polymer embedding or covalent attachment. For cellular applications, polymer embedding may result in potential leaching and cracking issues that could compromise probe stability; in contrast, covalent attachment of fluorophores provides a stable binding to the fibre surface, minimizing the likelihood of detachment [43,44].

**Figure 1:**
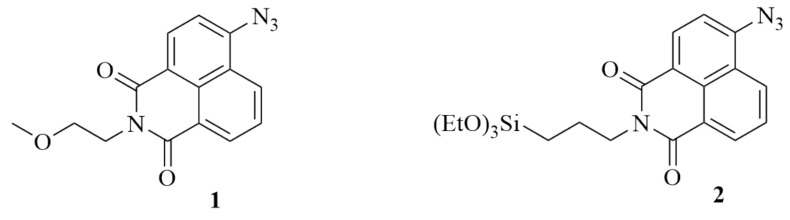
Chemical structures of probes **1** and **2**.

Here, we report the first demonstration of a covalently coated optical fibre sensor for HS^−^ in solution, to the best of our knowledge. We utilised the 4-azido-1,8-naphthalimide scaffold and integrated it with a highly sensitive fibre-optic platform to detect HS^−^ in aqueous media. Azide was selected due to its specific selectivity towards HS^−^ among relevant biological species, as substantiated by prior research studies [2,3,6,20]. In order to determine the impact of attaching the 1,8-naphthalimide to the fibre on its photophysical properties, we synthesised probe (**1**) containing a methoxy handle, acting as our control probe which was not embedded into the fibre and probe (**2**) with an APTES functionality, enabling covalent attachment of the probe to the optical fibre tip. The chemical structures of both probes are shown in Figure 1.

We evaluated the spectral response of **1** to HS^−^ using UV-Vis and fluorescence spectrophotometry to determine the suitable excitation wavelength and monitor the temporal evolution of the probe’s fluorescence emission, respectively. Subsequently, we coated the optical fibre tip with **2** and verified the presence and homogeneity of the coating across the fibre tip cross section using x-ray photoelectron spectroscopy (XPS). Once the fibre tips were coated, their ability to detect HS^−^ was investigated using a bespoke fibre-optic setup developed in-house, allowing us to compare the response with that obtained from the control probe. We then investigated the impact of pH on the spectral response of **2** coated on the optical fibre tip. The examination was conducted at pH 7.4 and 6.4, which closely resemble the pH conditions found in healthy and cancerous tissues, respectively [45,46].

## 2. Materials and Methods

### 2.1. General Experimental

All chemicals were purchased from commercial sources (Sigma-Aldrich, AK Scientific, Combi-Blocks, Alfa Aesar and Strem chemicals) with a reagent grade purity (95–99%), and used as received. ^1^H and ^13^C nuclear magnetic resonance (NMR) spectra were obtained using a Bruker AVANCE III HD 500 spectrometer at 298 K and were analysed using Bruker Topspin 3.2 software. Samples were dissolved in CDCl_3_ or dimethyl sulfoxide (DMSO)-*d*_6_ where specified, with the residual solvent peak used as the internal reference—CDCl_3_; 7.26 (^1^H) and 77.16 (^13^C) and DMSO-*d*_6_; 2.50 (^1^H) and 39.52 (^13^C) [47]. ^1^H NMR spectra are reported as follows: chemical shift δ, multiplicity (s = singlet, d = doublet, t = triplet, q = quartet, dd = doublet of doublets and m = multiplet), coupling constant (*J*, Hz) and assignment. A copy of all NMR spectra can be found in the Supplementary Materials (Figures S1–S8). High-resolution mass spectrometry (HRMS) spectra were recorded on an AB SCIEX TripleTOF 5600 mass spectrometer and ionisation of the samples was carried out using electrospray ionisation (ESI). The samples were prepared in 50:50 CH_3_CN/H_2_O solvent system containing 0.1% formic acid. Reactions performed using microwave irradiation were conducted in an Anton Paar Monowave 400 Microwave Reactor which has a maximum power output of 850 W. Melting points (m.p.) were determined using an ISG^®^ melting point apparatus and are uncorrected. Thin layer chromatography (TLC) was performed on Merck silica gel 60 F_254_ pre-coated aluminium plates (0.2 mm) and visualised using UV light (λ = 254 nm and 314 nm).

Absorption and emission spectra were recorded at room temperature using a Cary 50 UV-Visible (UV-Vis) spectrophotometer and a Varian Cary eclipse fluorescence spectrophotometer, respectively. The spectra were acquired using suprasil quartz cuvettes of 1 cm path length at right angle detection. We used an excitation slit width of 2.5 nm, an emission slit width of 5 nm, a PMT voltage set on high and a scan rate of 600 nm/min. Background correction was applied to all UV-Vis and fluorescence experiments using a solvent blank where relevant.

The multimode optical fibres used in this work, with 200/220 µm core/cladding diameter and 0.22 numerical aperture, were purchased from Thorlabs (FG200UEA). The optical fibres were cleaved using a Vytran LDC400 fibre cleaver. In situations necessitating the splicing of optical fibres, a Fujikura 90S splicer was employed. To detect laser power output throughout the experiments, a 2 GHz Si Photodetector (DET025AFC/M, from Thorlabs) was employed. A digital oscilloscope was utilised to record the laser pulses. All fluorescence spectra in the optical fibre setup were measured using a QEPro Ocean Optics spectrometer with SMA fibre connector.

### 2.2. Synthesis of the Fluorescent Probes

In this study, two fluorescent probes (**1** and **2**) were synthesised through a two-step process, as explained below.

#### 2.2.1. 6-Bromo-2-(2-methoxyethyl)-1H-benz[de]isoquinoline-1,3(2H)-dione (**4**)

In a 30 mL microwave vial, a mixture of 4-bromo naphthalic anhydride (**3**) (528 mg, 1.91 mmol), 2-methoxyethan-1-amine (0.33 mL, 3.91 mmol) and EtOH (5 mL) was irradiated at 100 °C for 1 h. The resulting mixture was diluted in H_2_O (20 mL), cooled to 0 °C and the solid was collected using vacuum filtration. After washing with cold H_2_O (60 mL), the desired product was isolated as a yellow solid (562 mg, 88%). m.p. 163–164 °C (lit. 162–163 °C) [48]. ^1^H NMR (500 MHz, CDCl_3_) δ 8.67 (1H, d, *J* = 7.3 Hz, H9), 8.57 (1H, d, *J* = 8.5 Hz, H7), 8.41 (1H, d, *J* = 7.9 Hz, H4), 8.03 (1H, d, *J* = 7.9 Hz, H5), 7.84 (1H, dd, *J* = 8.5, 7.3 Hz, H8), 4.41 (2H, t, *J* = 5.9 Hz, NCH_2_), 3.71 (2H, t, *J* = 5.9 Hz, OCH_2_), 3.35 (3H, s, OMe). ^13^C NMR (125 MHz, CDCl_3_) δ 163.88, 163.86, 133.5 (C7), 132.3 (C9), 131.5 (C4), 131.2 (C5), 130.8 (C6), 130.5 (C6a), 129.2 (C9b), 128.2 (C8), 123.2 (C9a), 122.3 (C3a), 69.7 (OCH_2_), 59.0 (OMe), 39.5 (NCH_2_). HRMS (ESI, *m/z*) for C_15_H_12_^79^BrNO_3_ [M + H]^+^ calc. 334.0073; found 334.0075.

#### 2.2.2. 6-Azido-2-(2-methoxyethyl)-1H-benzo[de]isoquinoline-1,3(2H)-dione (**1**)

A mixture of bromo (**4**) (108 mg, 0.323 mmol), NaN_3_ (108 mg, 1.66 mmol) and *N,N*-Dimethylformamide (DMF) (2 mL) was protected from light and stirred at 80 °C for 3 h. The reaction vessel was cooled to ambient temperature, diluted in EtOAc (20 mL) and transferred to a separatory funnel. The organic phase was washed with H_2_O (3 × 20 mL), brine (20 mL), then dried (Na_2_SO_4_), filtered and concentrated under reduced pressure to give the product as a dark orange solid (80 mg, 84%). m.p. 130–132 °C. ^1^H NMR (500 MHz, CDCl_3_) δ 8.65 (1H, d, *J* = 7.3 Hz, H9), 8.59 (1H, d, *J* = 8.0 Hz, H4), 8.44 (1H, d, *J* = 8.9 Hz, H7), 7.74 (1H, dd, *J* = 8.9, 7.3, Hz, H8), 7.47 (1H, d, *J* = 8.0 Hz, H5), 4.43 (2H, t, *J* = 5.9 Hz, NCH_2_), 3.73 (2H, t, *J* = 5.9 Hz, OCH_2_), 3.39 (3H, s, OMe). ^13^C NMR (125 MHz, CDCl_3_) δ 164.5, 164.0, 143.9 (C6), 132.7 (C9), 132.2 (C4), 129.6 (C6a), 129.2 (C7), 127.2 (C8), 124.7 (C9b), 122.9 (C9a), 119.2 (C3a), 115.0 (C5), 70.0 (OCH2), 59.1 (OMe), 39.6 (NCH_2_). HRMS (ESI,*m/z*) for C_15_H_12_N_4_O_3_ [M + H]^+^ calc. 319.0802; found 319.0802. NMR data matched those reported in the literature [49]. This compound was used as is for future experimental/optical evaluations.

#### 2.2.3. 6-Bromo-2-(3-(triethoxysilyl)propyl)-1H-benzo[de]isoquinoline-1,3(2H)-dione (**5**)

In a 30 mL microwave vial, a mixture of 4-bromo naphthalic anhydride (**3**) (556 mg, 2.0 mmol), APTES (0.90 mL, 3.85 mmol) and EtOH (5 mL) was irradiated at 100 °C for 2 h. The resulting mixture was diluted in H_2_O (20 mL), cooled to 0 °C and the solid was collected using vacuum filtration. After washing with cold H_2_O (100 mL) the desired product was isolated as a dark yellow solid (793 mg, 83%). m.p. 127–128 °C (lit 128–130 °C) [50]. ^1^H NMR (500 MHz, CDCl_3_) δ 8.65 (1H, d, *J* = 7.3 Hz, H9), 8.57 (1H, d, *J* = 8.5 Hz, H7), 8.41 (1H, d, *J* = 7.9 Hz, H4), 8.04 (1H, d, *J* = 7.9 Hz, H5), 7.84 (1H, dd, *J* = 8.5, 7.3 Hz, H8), 4.16 (2H, t, *J* = 7.5 Hz, NCH_2_), 3.81 (6H, q, *J* = 7.0 Hz, 3 × OC*H_2_*CH_3_), 1.88–1.81 (2H, m, NCH_2_C*H_2_*), 1.21 (9H, t, *J* = 6.8 Hz, 3 × OCH_2_C*H_3_*), 0.75 (2H, t,*J* = 8.7 Hz, SiCH_2_). ^13^C NMR (125 MHz, CDCl_3_) δ 163.72, 163.70, 133.4 (C7), 132.2 (C9), 131.4 (C4), 131.2 (C5), 130.8 (C6), 130.3 (C6a), 129.2 (C8), 128.2 (C9a), 123.3 (C3a), 122.5 (NCH2), 122.5 (O*C*H_2_CH_3_), 43.1 (NCH_2_*C*H_2_), 21.6 (OCH_2_*C*H_3_), 8.1 (SiCH_2_). HRMS (ESI, *m/z*) for C_15_H_26_^79^BrNO_5_Si [M + Na]^+^ calc. 502.0656; found 502.0653.

#### 2.2.4. 6-Azido-2-(3-(triethoxysilyl)propyl)-1H-benzo[de]isoquinoline-1,3(2H)-dione (**2**)

A mixture of bromo (**5**) (219 mg, 0.46 mmol), NaN_3_ (174 mg, 2.68 mmol) and DMF (3 mL) was protected from light and stirred at 80 °C for 4 h. The reaction vessel was cooled to ambient temperature, diluted in EtOAc (20 mL), cooled to 0 °C and the resulting solid was collected using vacuum filtration. After washing with cold H_2_O (100 mL) the desired product was isolated as an orange solid (125 mg, 62%). m.p. 123–126 °C. ^1^H NMR (500 MHz, DMSO-*d*_6_) δ 8.52 (1H, d,*J* = 7.3 Hz, H9), 8.47 (1H, d, *J* = 8.0 Hz, H4), 8.41 (1H, d, *J* = 8.4 Hz, H7), 7.85 (1H, dd, *J* = 8.4, 7.3 Hz, H8), 8.0 (1H, d, *J* = 8.0 Hz, H5), 4.00 (2H, t, *J* = 7.6 Hz, NCH_2_), 3.73 (6H, q, *J* = 7.0 Hz, 3 × OC*H_2_*CH_3_), 1.73–1.64 (2H, m, NCH_2_C*H_2_*), 1.12 (9H, t, *J* = 7.0 Hz, 3 × OCH_2_C*H_3_*), 0.63 (2H, t, *J* = 8.6 Hz, SiCH_2_). ^13^C NMR (125 MHz, DMSO-*d*_6_) δ 163.2, 162.8, 142.8 (C7), 131.6 (C9), 131.5 (C5), 128.3 (C4), 127.3 (C8), 123.5 (C3a), 122.2 (C6a), 118.2 (C9b), 116.0 (C9a), 57.7 (C6), 42.2 (NCH_2_), 21.0 (O*C*H_2_CH_3_), 18.2 (NCH_2_*C*H_2_), 8.9 (SiCH_2_), 7.5 (OCH_2_*C*H_3_). HRMS (ESI, *m/z*) for C_15_H_26_N_4_O_5_Si [M + Na]^+^ calc. 465,1565; found 465.1563. NMR data matched those reported in the literature [51]. This compound was further subjected to fibre coating.

### 2.3. Surface Functionalisation and Coating of the Optical Fibres

**Figure 2:**
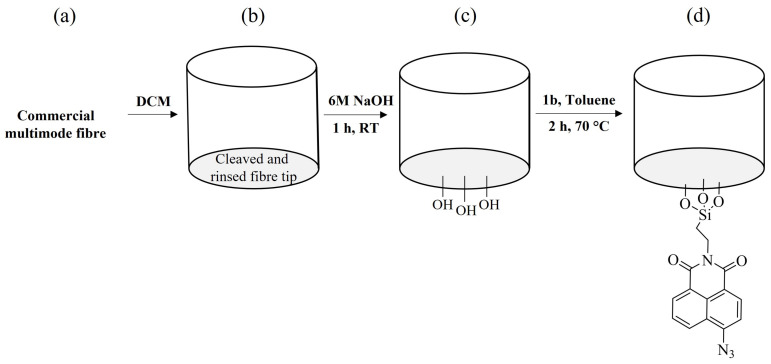
Optical fibre coating process. (**a**) The polymer coatings of commercial multimode optical fibre were removed using dichloromethane (DCM), (**b**) the distal ends of the optical fibre were cleaved and rinsed with ethanol, (**c**) they were immersed in 6M NaOH for 1 h at room temperature (RT) and (**d**) immersed in the coating solution containing **2** dissolved in toluene for 2 h at 70 °C.

The optical fibre tips were coated with **2** following multiple steps which are schematically demonstrated in Figure 2.

The acrylate coating of the optical fibres was removed with DCM. The distal ends of the optical fibres were cleaved and rinsed with pure ethanol. The freshly cleaved distal ends were immersed in 6M NaOH for 1 h to activate the hydroxyl groups at the optical fibre tip for covalent bonding [52,53,54], which was conducted in a sealed environment. Afterwards, the optical fibres were rinsed thoroughly with MilliQ water and left to dry at room temperature. In the subsequent step, the optical fibres were inserted into a glass tube containing a 0.22 mM solution of **2** in anhydrous toluene. This solution was prepared as follows; **2** was dissolved in DMSO to prepare a 22.59 mM stock solution which was diluted in 10 mL anhydrous toluene to 0.22 mM final solution with less than 1% DMSO obtained. To prevent ambient humidity, the tube was filled with nitrogen gas [55] and a septa with needle was used to cap the tube (the tube is shown in the Supplementary Materials Figure S9). The fibres and solution were heated at 70 °C for 2 h. This moderate temperature was used to decrease the number of molecules bonded weakly to the surface via hydrogen bonding, enhancing the quality of the coating [55,56]. Upon completion of the coating process, the optical fibres were thoroughly rinsed with an ample amount of MilliQ water and dried under nitrogen gas. All coated optical fibres were tested on the same day of coating.

### 2.4. X-ray Photoelectron Spectroscopy

Surface characterisation of the fluorophore-coated optical fibres, along with negative control samples, was performed with XPS, KRATOS AXIS SUPRA+, with the capability of parallel imaging to provide lateral distribution of key elements at the surface. The aim of XPS was to confirm the coating and evaluate the uniformity of the coating across the optical fibre tip surface and consistency of the coating among multiple optical fibres. Two types of fibres were examined via XPS, control and coated. Coated optical fibres were prepared as per detailed in Section 2.3 above. Control fibres were subject to the same process; however, in the step of inserting the fibres in the coating solution, they were only soaked in DMSO (1%) and toluene (99%) and the fluorophore was omitted at that stage. XPS measurements were performed in a chamber with an ultra high vacuum, $2\times 10^{-9}$ Torr. All samples were excited with Al monochromatic Kα (energy 1486.6 eV) source. The output power of the X-ray source was set to be 225 W with 15 kV voltage and 15 mA current. Wide and narrow scan spectra were recorded with pass energy of 160 eV and 20 eV, respectively. The instrument allows the analysis of the surface composition within the first 10 nm. For high quality chemical and elemental analysis, 110 μm × 110 μm area of the optical fibre tip was selected.

All data from the XPS were analysed with CasaXPS software, version 2.3.25PR1.0 (Casa Software Ltd., Wilmslow, Cheshire, UK). All found chemical elements were charge corrected to adventitious hydrocarbon peak (284.8 eV) [57]. Following the identification of the regions associated with each chemical element, the Shirley background was selected and the corresponding proportional background was subsequently subtracted from each peak. Then, the calculation of atomic percentage of the elements, high-resolution analysis, peak fitting were performed using the software. In each high-resolution spectrum, all components were restricted to possessing the same full width at half maximum, which is a prevalent approach and has been previously documented [58,59]. The utilised peak shape model was a Gaussian/Lorentzian product function (G/L = 30).

### 2.5. Spectral Measurements of Free Fluorophore in Solution

**1** was dissolved in DMSO to prepare a 9.8 mM stock solution, which was diluted in phosphate buffer solution (PBS, pH 7.4) to 0.25 mM to ensure the final solution had less than 1% DMSO present. DMSO was used to ensure full solubility. A 10 mM sodium hydrosulphide (NaSH) solution was prepared by dissolving solid NaSH in PBS solution (pH 7.4). The absorption measurements of **1** in response to HS^−^ were conducted using a UV-Vis spectrophotometer within the wavelength range of 320–800 nm. Following the addition of three equivalents of NaSH to the dissolved **1** in DMSO within a cuvette, the absorption spectra were measured over 30 min at 3 min time intervals. The choice of 405 nm as the excitation wavelength was determined by the compound’s absorption spectra, wherein both azide and amine exhibited very similar absorption levels at this specific wavelength, which can be considered a pseudo-isosbestic point. The same procedure was followed to measure the changes in the fluorescence signal of the fluorophore using the fluorimeter, with an excitation wavelength of 405 nm and a wavelength range of 410 to 800 nm.

### 2.6. Optical Fibre-Based Sensing Setup

**Figure 3:**
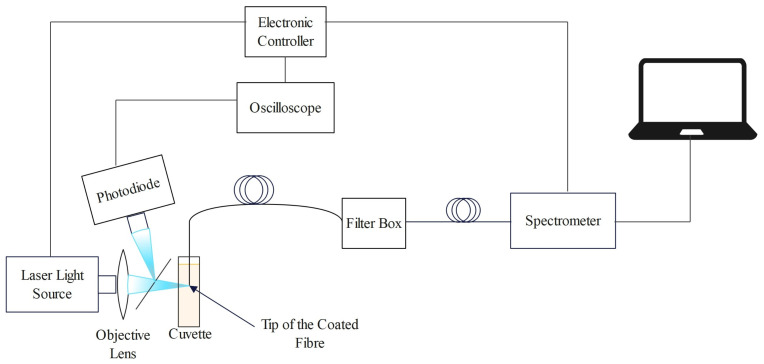
Optical fibre-based sensing set-up. Excitation light was focused to the tip of the optical fibre using an objective lens. The generated fluorescence emission was captured by the optical fibre and delivered to the spectrometer after passing through long pass filters in the filter box. The photodiode and oscilloscope were used to detect and record the laser power partially reflected by a glass slide during the experiment, respectively. An electronic controller was built and programmed to trigger both the light source and the spectrometer.

The developed optical-fibre setup is schematically shown in Figure 3.

The excitation light with a wavelength of 405 nm emitting from a laser light source was focused to the tip of the coated optical fibre using an objective lens placed close to the laser output aperture. In this configuration, the fluorophores at the tip were excited from the side, which minimises the autofluorescence from the optical fibre. In the excitation light path, a glass slide was employed to reflect a small portion of the light toward the photodetector which was connected to a digital oscilloscope to record the laser output power during the experiment. By continuously monitoring the laser power, we could measure the fluctuations in laser power throughout the experiments. This enabled us to determine whether the variations in laser power could affect the measurement of fluorescence intensity of the fluorophore. The tip of the coated optical fibre vertically mounted in a quartz cuvette, as a test solution container, was aligned to be at the focal point of the lens for excitation of the fluorophores. The coated optical fibre was spliced to a multimode patch cable with the same properties to deliver the fluorescence signal to the spectrometer. In the path to the spectrometer, long pass filters were used to avoid the excitation wavelength. An electronic controller was also designed and programmed to trigger the laser source to emit excitation pulses with 100 ms integration time and trigger the spectrometer with the same integration time to collect the fluorescence signal.

For the experiment, the coated fibre tip was inserted into a cuvette containing 2 mL solution of PBS (pH 7.4) and left to calibrate. Immediately prior to adding HS^−^, several excitation pulses were used to capture the background fluorescence emission. Then HS^−^ was added directly to the cuvette and the fluorescence intensity was measured over time. The concentration of HS^−^ in 2 mL PBS for all the experiments was 10 mM. After each experiment, the optical fibre was replaced as the chemical reaction between the fluorophore and HS^−^ was not reversible. The detection of HS^−^ was performed in PBS solution with two different pHs, 7.4 and 6.4, to investigate the effect of pH on the sensitivity of the fluorophore. The above protocol was repeated for pH 6.4, where the pH of PBS solution was altered to 6.4 by adding HCl 37% to PBS with pH 7.4.

## 3. Results and Discussion

### 3.1. Synthesis

**Scheme 1:**
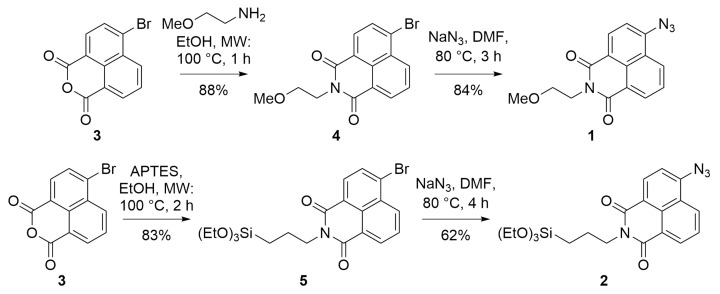
Synthesis of 4-azido-1,8-naphthalimide probes **1** and **2**.

To establish if attaching the 1,8-naphthalimide to the fibre altered the photophysics, we synthesised two compounds: one with a methoxy handle as the control and another with the APTES handle, which would allow coupling to the fibre surface. The methoxy also allowed for evaluation of photophysics in glass cuvettes. The APTES derivative, as expected, reacted slowly with the glass altering the results. Hence, **1** was used in solution for cuvette measurements and **2** was coupled to the fibre. The synthesis of both **1** and **2** was conducted in two steps. Firstly, 4 bromo naphthalic anhydride (**3**) was reacted with either 2-methoxyan-1-amine or APTES to give 1,8-naphthalimides **4** and **5**, respectively (Scheme 1). Nucleophilic aromatic substitution with sodium azide then gave 4-azido derivatives **1** and **2** in good yields. The synthetic scheme is as shown in the Scheme 1.

### 3.2. Coating of Optical Fibres and Characterisation

**Figure 4:**
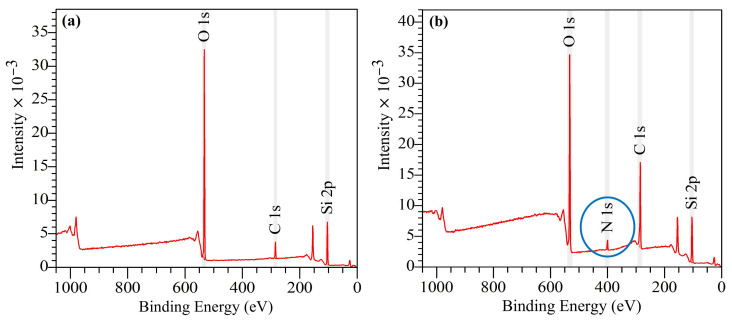
XPS spectrum of (**a**) control and (**b**) coated optical fibres. Main chemical elements of both samples are labelled. In (**b**), a blue circle indicates the N 1s peak, attributed to the azide group.

With **2** in hand, covalent attachment to the fibre tip was pursued. This process was carried out following the detailed procedure outlined in Section 2.3. In summary, the cleaved and rinsed optical fibres were first hydroxylated using NaOH and then they were rinsed with MilliQ water and dried at room temperature. Subsequently, the fibres were immersed in a coating solution that contained **2** dissolved in anhydrous toluene. When the coating was complete, the optical fibres were rinsed and dried using nitrogen gas. To ensure the presence of the coating on the fibre tips, XPS survey and high-resolution scans of both control and coated optical fibres were performed. For control samples, a similar treatment was applied; however, the coating solution did not contain **2**. Wide survey scans demonstrated the presence of C 1s (284.8 eV), O 1s (532.8 eV) and Si 2p (103.8 eV) elements on both fibres (Figure 4a for control and Figure 4b for coated fibres). Importantly, it is only the coated fibre where N 1s (399.8 eV) could be observed (Figure 4b). The noticeable N 1s peak (blue circle in Figure 4b) with 3.15 ± 0.18 atomic percentage observed in the spectrum of the coated optical fibres corresponded to the azide group of the fluorophore which was absent in the control samples. This suggests that the coating was formed at the fibre tip.

Table 1 presents the atomic concentration percentage of individual chemical elements in both control and coated optical fibres, averaged over multiple samples (four coated and two control), along with their corresponding standard deviation values.

The carbon peak observed in the control samples can be attributed to adventitious carbon contamination [60,61] and the solvents used in the process of preparing samples, since silica optical fibre itself does not contain any carbon molecules. Importantly, the percentage of carbon on the coated fibre was higher than in the control; this may be attributed to the carbon structure of the fluorophore on the coated fibre. Moreover, it was found that the Si 2p and O 1s peaks originating from the surface of the control samples (SiO_2_) exhibited attenuation in the coated optical fibres. This attenuation can be attributed to the coverage of the optical fibre by the coating [62], which potentially impeded the detection of silica and oxygen signals from the optical fibres.

**Figure 5:**
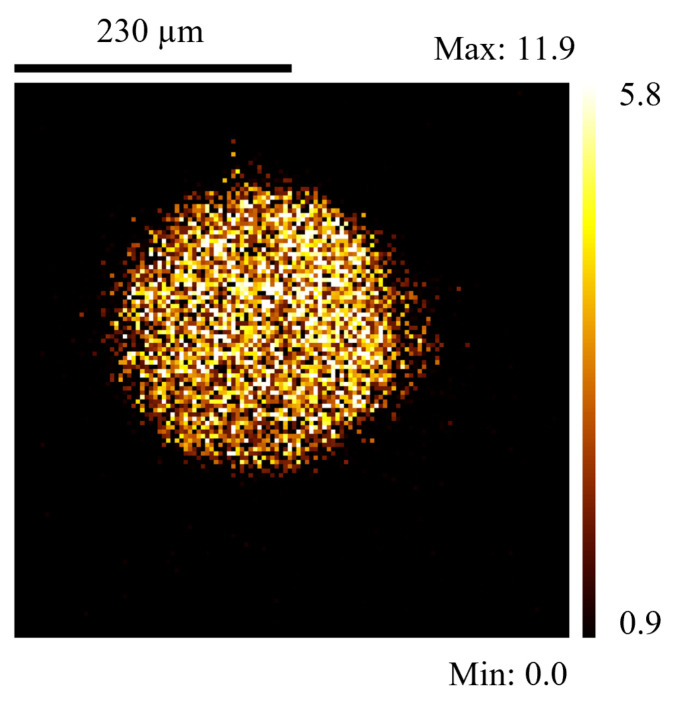
Nitrogen distribution over the surface of a coated optical fibre. The light yellow regions have higher concentration of nitrogen when compared to the black areas.

The cross-sectional images obtained from the XPS of the fluorophore-coated optical fibres unveiled a relatively uniform distribution of chemical elements (C, O, N and Si) across the optical fibre tip. The even distribution of nitrogen, which serves as a crucial chemical element representing the fluorophore, is illustrated as an example in Figure 5. The relatively homogeneous distribution of nitrogen provides evidence of the cross-sectional coverage of the optical fibre tip in a near-uniform manner.

The high-resolution (HR) XPS spectra of both control and coated optical fibres are presented in Supplementary Materials Figures S10 and S11, respectively. Table 2 summarises the averaged curve-fitting data with standard deviation for carbon, silica and oxygen peaks of both control and coated optical fibres.

The HR XPS spectra for a range of chemical elements involving C, O and Si supported the earlier findings. As expected, both control and coated fibres showed the evidence of C-C/C-H, C-O, C=O, Si-O-Si and SiO_2_ bonds. As discussed earlier, the presence of carbon bonds on the control fibre was likely due to the atmospheric carbon and the use of organic solvents; however, on the coated fibre, it could be attributed a combination of solvent and probe. The Si-O bonds may arise from either the APTES covalent bonding to the surface or the inherent chemical structure of the optical fibre. The SiO_2_ bond percentage in the Si 2p peak did decrease in the coated fibre compared with control and this may be due to the addition of the coating on top of the fibre. The Si 2p spectrum of the coated fibre did show a peak at the right shoulder (Supplementary Materials Figure S11) which aligns with the Si-C bond. The presence of this bond, which was absent in the control fibres, confirms the covalent attachment of **2** to the silica fibre. Unfortunately, an appropriate HR curve fitting of nitrogen peak could not be performed with a high level of certainty due to its small peak and the only observable peak corresponded to the N-C bond (Supplementary Materials Figure S11). The presence of two chemical bonds, C-N and N-C=O, in the carbon and oxygen peaks of the coated samples provided evidence of coating at the optical fibre tip. The relatively low standard deviation of each chemical bond in Table 2 implies that the chemical composition of the coating remains relatively consistent across different optical fibre samples, indicating an acceptable degree of reproducibility in the coating process.

### 3.3. Spectral Response of Free Fluorophore in Solution

**Figure 6:**
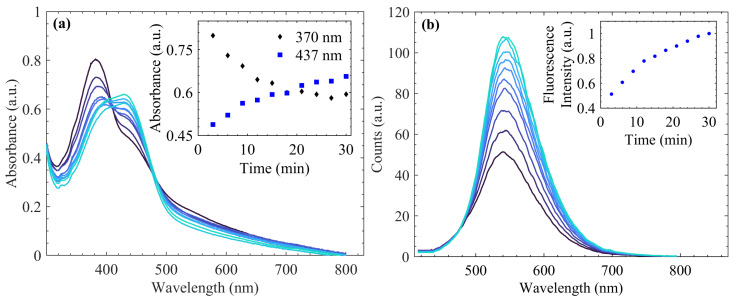
(**a**) The absorption spectra of **1** following the addition of three equivalents of HS^−^ over a period of 30 min, (inset) the time-dependent changes in peaks at 370 and 437 nm. (**b**) Emission spectra of **1** ($\lambda_{exc}$ = 405 nm) after adding three equivalent HS^−^, (inset) normalised time-dependent behaviour of the fluorescent peak at 540 nm over 30 min reaction time. The colour gradient ranging from dark to bright blue indicates the (**a**) absorption and (**b**) emission spectral changes over time.

The absorption spectra of **1** were collected using UV-Vis spectrophotometery. Ideally, optical measurements would be performed on **2** to determine the optimum excitation wavelength and to explore the effect of attachment to a fibre on optical properties. However, due to the APTES functionality, **2** was found to react with glass. Therefore, **1** was employed as a control. The changes of the absorption spectra in response to three equivalents of HS^−^ are shown in Figure 6a.

Prior to the addition of HS^−^, a primary absorption peak at approximately 380 nm was observed ($\pi \to \pi^{*}$ transition). This can be attributed to the presence of the naphthalene moiety [20]. Upon the addition of HS^−^, the peak decreased in intensity (Figure 6a inset, black diamonds). Concurrently, a shoulder around 437 nm ($n\to \pi^{*}$ transition) appeared which increased in absorption (Figure 6a inset, blue squares). It is hypothesised that the increase in the secondary peak absorption is derived from the amine-naphthalimide compound formed as a result of the reaction with HS^−^ [27]. This finding was consistent with observations by Choi et al; however, it is important to note that their experiment involved using significantly larger quantities of HS^−^, with the concentration increasing over time, unlike our time-monitoring approach [20]. In this study, 405 nm was chosen as the excitation wavelength where both azide and amine have very similar absorption levels.

After finding the optimum excitation wavelength, the fluorescence response of **1** was measured after adding HS^−^. Figure 6b displays the fluorescence-emission spectra of **1** in PBS (pH 7.4) using 405 nm excitation and three equivalents of HS^−^ over a period of 30 min. Upon the addition of HS^−^ to the fluorophore solution, a marked increase in fluorescence intensity was observed, indicating the effectiveness of the fluorophore as a potential probe for HS^−^ detection. The temporal evolution of the fluorescence peak at around 540 nm is illustrated in Figure 6b inset. The obtained data demonstrate that the addition of HS^−^ resulted in a more than two-fold enhancement of the emission intensity. This is also consistent with the previously reported naphthalimide azide HS^−^ sensors [20,27]. Furthermore, the fluorophore exhibited a large Stokes shift, with a wavelength distance between excitation and emission peaks of approximately 170 nm. This facilitated the elimination of excitation light using long-pass filters in the optical fibre-based setup.

### 3.4. Detection of Hydrosulphide Ion with the Optical Fibre-Based Sensing Setup

After characterizing the response of **1** in solution to HS^−^, the subsequent step involved investigating whether the response would be modified by coupling the identical napthalimide azide aromatic structure to the fibre through the APTES linker. To accomplish this, the coated fibre was used in the developed fibre optic setup, initial baseline recordings were obtained and subsequently, HS^−^ was added to measure the resulting fluorescence change. We found that the changes in fluorescence intensity, with a peak at 545 nm, were similar to what we observed with **1**, exhibiting an increase in the intensity when subjected to HS^−^.

**Figure 7:**
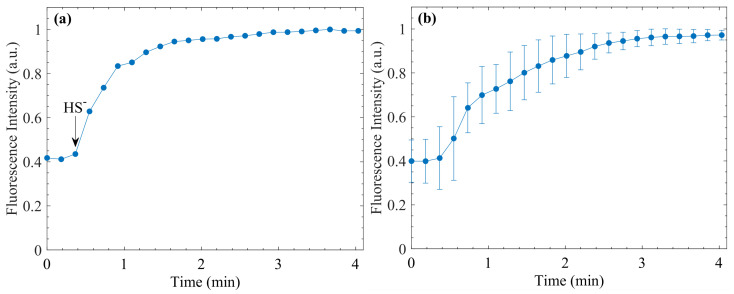
(**a**) The temporal change of the normalised fluorescence intensity of a coated optical fibre in one experiment before and after HS^−^ was added. The black arrow indicates the time HS^−^ was added to the PBS in cuvette. (**b**) The average of the normalised fluorescence intensity variation obtained from five experiments. The error bars represent the standard deviation around the mean value. Dots are experimental results and the line is connecting the experimental points.

Figure 7 shows that the response time, which is considered as the time it takes for the fluorescence intensity to reach 90% of its maximum value, is around 2 min. The fluorescence intensity shows a lower rate of increase around 3–4 min time, which indicates the stabilisation of the reaction between fluorophore **2** and HS^−^ completed within 4 min (Figure 7a). The fluorescence response is depicted through the integration of emission intensities within a specified range. Interestingly, the response rate for **2** coupled to the fibre was considerably faster than that observed for **1** in solution. While **2** reached a plateau in just 4 min, it took approximately 30 min for **1** to reach equilibrium. This could be attributed to the lower number of fluorophores attached to the fibre in the case of **2**; however, for biological sensing applications, a rapid response like that of **2** holds greater advantages over the 30 min response seen in **1**. Since the reduction of azide to amine is not reversible [63], each experiment required the utilization of a new fibre. Consequently, in order to assess reproducibility, these experiments were repeated on the same day using distinct fibres, and the average plot is illustrated in Figure 7b. A slight deviation is noted, but overall the result was reproducible. The deviation from the average plot may be attributed to the slight changes in optical setup and the coating between each fibre. Throughout the experiments, the laser power output was continually monitored and found to exhibit less than 1% variability. However, the amount of excitation light delivered to the fluorophores at the optical fibre tip depended on the precise positioning of the tip at the focal point of the objective lens. Nevertheless, we assumed that when the reaction between the fluorophore and HS^−^ was completed at 4 min, the emission intensity is proportional to the excitation; therefore, we normalised the emission intensity to the final value at 4 min. Moreover, while the XPS showed that there were minimal differences between coated fibres, differences were still noted.

### 3.5. pH Effects on the Probe Response

The effects of pH on azide-containing napthalimide-based fluorophores have been extensively researched [4,27,64,65]. Hence, it was important to examine the impact of pH on the response in our study once coated to the fibre. In this study, two specific pH levels were selected, pH 7.4 which corresponds to the physiological pH of biological tissues and pH 6.4 which represents the pH typically associated with cancerous tissues [45,46]. By examining the fluorescence response at these pHs, the potential applicability of the probe in detecting and differentiating healthy and cancerous tissues can be assessed.

**Figure 8:**
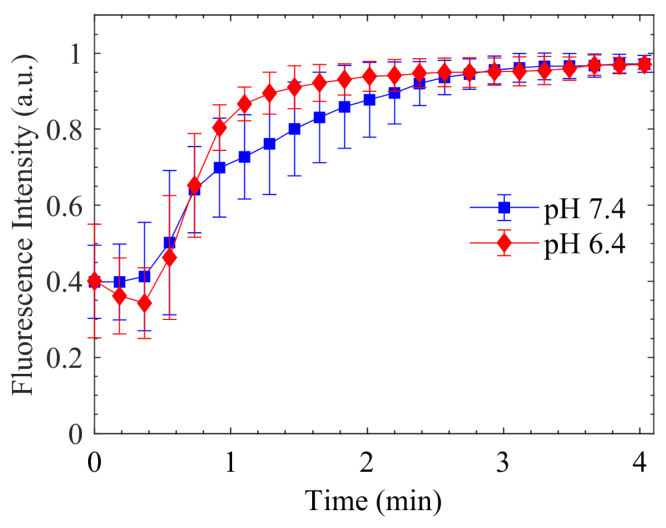
Normalised fluorescence intensity changes of the fluorophore-coated optical fibre in response to HS^−^ dissolved in PBS with pH value of 7.4 and 6.4, averaged over multiple experiments. Dots are experimental results and the line is connecting the experimental points.

The experimental procedure for pH 6.4 was identical to that employed for pH 7.4. Here, a new batch of coated optical fibres was prepared and employed on the same day. The enhancement in fluorescence intensity upon the introduction of HS^−^ with an approximately similar pattern as for pH 7.4 was observed. A comparison of the experimental results for pH 7.4 and 6.4 (Figure 8) reveals an overlap between the two curves when considering the error bars, with only slight discrepancies. These variations can likely be attributed to several factors, including the use of different coated optical fibres for each experiment and the level of excitation power reaching the fluorophores at the tip of the optical fibre in each experiment. Based on the findings presented in the research works [4,27,64,65], the naphthalimde azide-containing fluorophore is reported to exhibit a decrease in fluorescence intensity when the pH of the test solution became acidic. However, our findings indicate that the fluorescence response of the fluorophore coated on the fibre tip remains unaffected when the pH of the test solution decreased to 6.4. This implies that by attaching the probe to the fibre, it has the potential to be utilised for the detection of HS^−^ at cancerous pH levels.

## 4. Conclusions

This paper presents the synthesis and characterization of 1,8-naphthalimide-based fluorescent probes with an azide group, as an HS^−^ sensitive agent, for the detection of HS^−^ in aqueous media. A control 1,8-naphthalimide compound **1** which was amenable to UV-Vis and fluorimetry experiments, revealed the correct excitation wavelength and its time-dependent response to HS^−^. To assess the fluorescence-emission behavior of the fluorophore when immobilised on a surface, optical fibres were coated with the fluorophore containing an APTES group and subsequently utilised in a developed optical fibre-based setup. The time-dependent evolution of fluorescence intensity exhibited a notable increase within an approximately 2 min timeframe, followed by a stabilisation for the rest of the experiment. The fluorophore-coated optical fibres were examined at pH 7.4 and 6.4 in order to determine any potential pH dependency in this range. The findings showed that the fluorescence emissions were consistent for the two pHs, which is important for cellular experiments. Surface characterization of the control and coated optical fibres was carried out using XPS. The XPS analysis confirmed the presence of the fluorophore at the optical fibre tip, as indicated by the nitrogen peak in the coated optical fibre survey, along with the detection of C-N and N-C=O bonds through carbon and oxygen high-resolution curve fitting. Furthermore, the XPS results demonstrated the reproducibility of the coating process and the uniform coverage of the optical fibre tip. Currently, ongoing optimization of the optical fibre-based sensing setup is in progress, with the ultimate aim of designing a sensor capable of detecting HS^−^ in biological samples.

## Figures and Tables

**Table 1:** Main chemical element percentage for control and coated optical fibres from XPS data.

	C 1s (%)	O 1s (%)	N 1s (%)	Si 2p (%)
Control optical fibre	13.91 ± 1.53	55.75 ± 1.09	0	30.33 ± 0.43
Coated optical fibre	45.05 ± 5.85	31.93 ± 3.90	3.15 ± 0.18	19.86 ± 1.84

**Table 2:** Average percentage contribution of C 1s, O 1s and Si 2p high resolution peaks.

Element	Chemical Bond Contribution	Coated (%)	Control (%)
C 1s	C-C/C-H	77.36 ± 4.37	89.18 ± 1.58
	C-O/C-N	14.64 ± 2.70	8.62 ± 1.09
	C=O	7.99 ± 1.81	2.19 ± 0.48
O 1s	Si-O-Si	91.35 ± 2.51	97.26 ± 0.19
	O-C	0.88 ± 0.65	2.74 ± 0.19
	N-C=O	7.74 ± 1.58	0
Si 2p	SiO_2_	79.18 ± 2.88	100
	Si-C	20.81 ± 2.88	0

## Data Availability

The data presented in this study are available on request from the corresponding author.

## Supplementary Materials

The following supporting information can be downloaded at: https://www.mdpi.com/article/10.3390/bios13110949/s1: Figure S1: 1H NMR (500 MHz) spectra of 4 in CDCl3; Figure S2: 13C NMR (125 MHz) spectra of 4 in CDCl3; Figure S3: 1H NMR (500 MHz) spectra of 1 in in CDCl3; Figure S4: 13C NMR (125 MHz) spectra of 1 in in CDCl3; Figure S5: 1H NMR (500 MHz) spectra of 5 in in CDCl3; Figure S6: 13C NMR (125 MHz) spectra of 5 in CDCl3; Figure S7: 1H NMR (500 MHz) spectra of 2 in DMSO-d6; Figure S8: 13C NMR (125MHz) spectra of 2 in in DMSO-d6; Figure S9: Coating setup. A glass tube containing the coating solution with optical fibres; Figure S10: High resolution spectra of C 1s, O 1s, Si 2p and N 1s of control fibres; Figure S11: High resolution spectra of C 1s, O 1s, Si 2p and N 1s of coated fibres.

## References

[1] Usha S.P., Mishra S.K., Gupta B.D. (2015). Fibre optic hydrogen sulfide gas sensors utilizing ZnO thin film/ZnO nanoparticles: A comparison of surface plasmon resonance and lossy mode resonance. Sens. Act. B Chem..

[2] Yoo S.Y., Gopala L., Kang C., Lee M.H. (2022). Hydrogen sulfide-activatable fluorescence turn-on azide-containing naphthalimide derivative. Bull. Korean Chem. Soc..

[3] Lippert A.R., New E.J., Chang C.J. (2011). Reaction-based fluorescent probes for selective imaging of hydrogen sulfide in living cells. J. Am. Chem. Soc..

[4] Cha Y., Gopala L., Lee M.H. (2023). A bio-friendly biotin-coupled and azide-functionalized naphthalimide for real-time endogenous hydrogen sulfide analysis in living cells. Spectrochim. Acta Part A Mol. Biomol. Spectrosc..

[5] Zhang L., Li S., Hong M., Xu Y., Wang S., Liu Y., Qian Y., Zhao J. (2014). A colorimetric and ratiometric fluorescent probe for the imaging of endogenous hydrogen sulphide in living cells and sulphide determination in mouse hippocampus. Org. Biomol. Chem..

[6] Qiao Q., Zhao M., Lang H., Mao D., Cui J., Xu Z. (2014). A turn-on fluorescent probe for imaging lysosomal hydrogen sulfide in living cells. RSC Adv..

[7] Feng X., Zhang T., Liu J.T., Miao J.Y., Zhao B.X. (2016). A new ratiometric fluorescent probe for rapid, sensitive and selective detection of endogenous hydrogen sulfide in mitochondria. Chem. Commun..

[8] Liu D., Hessler W., Henary M. (2023). H_2_S Sensors: Synthesis, Optical Properties, and Selected Biomedical Applications under Visible and NIR Light. Molecules.

[9] Khattak S., Rauf M.A., Khan N.H., Zhang Q.Q., Chen H.J., Muhammad P., Ansari M.A., Alomary M.N., Jahangir M., Zhang C.Y. (2022). Hydrogen sulfide biology and its role in cancer. Molecules.

[10] Wang R.H., Chu Y.H., Lin K.T. (2021). The hidden role of hydrogen sulfide metabolism in cancer. Int. J. Mol. Sci..

[11] Shackelford R.E., Mohammad I.Z., Meram A.T., Kim D., Alotaibi F., Patel S., Ghali G.E., Kevil C.G. (2021). Molecular functions of hydrogen sulfide in cancer. Pathophysiology.

[12] Singh N., Sharma S., Singh R., Rajput S., Chattopadhyay N., Tewari D., Joshi K.B., Verma S. (2021). A naphthalimide-based peptide conjugate for concurrent imaging and apoptosis induction in cancer cells by utilizing endogenous hydrogen sulfide. Chem. Sci..

[13] Akbari M., Sogutdelen E., Juriasingani S., Sener A. (2019). Hydrogen sulfide: Emerging role in bladder, kidney, and prostate malignancies. Oxidative Med. Cell. Longev..

[14] Furne J., Saeed A., Levitt M.D. (2008). Whole tissue hydrogen sulfide concentrations are orders of magnitude lower than presently accepted values. Am. J. Physiol. Regul. Integr. Comp. Physiol..

[15] Duc C., Boukhenane M.L., Wojkiewicz J.L., Redon N. (2020). Hydrogen sulfide detection by sensors based on conductive polymers: A review. Front. Mater..

[16] Lawrence N.S., Davis J., Jiang L., Jones T.G., Davies S.N., Compton R.G. (2000). The electrochemical analog of the methylene blue reaction: A novel amperometric approach to the detection of hydrogen sulfide. Electroanalysis.

[17] Ali F.I., Awwad F., Greish Y.E., Mahmoud S.T. (2018). Hydrogen sulfide (H_2_S) gas sensor: A review. IEEE Sens. J..

[18] Pandey S.K., Kim K.H., Tang K.T. (2012). A review of sensor-based methods for monitoring hydrogen sulfide. TrAc Trends Anal. Chem..

[19] Allsop T., Neal R. (2021). A review: Application and Implementation of optic fibre sensors for gas detection. Sensors.

[20] Choi S.A., Park C.S., Kwon O.S., Giong H.K., Lee J.S., Ha T.H., Lee C.S. (2016). Structural effects of naphthalimide-based fluorescent sensor for hydrogen sulfide and imaging in live zebrafish. Sci. Rep..

[21] Yu F., Han X., Chen L. (2014). Fluorescent probes for hydrogen sulfide detection and bioimaging. Chem. Commun..

[22] Peng H., Cheng Y., Dai C., King A.L., Predmore B.L., Lefer D.J., Wang B. (2011). A fluorescent probe for fast and quantitative detection of hydrogen sulfide in blood. Angew. Chem..

[23] Elsayed S., de la Torre C., Santos-Figueroa L.E., Marin-Hernandez C., Martinez-Manez R., Sancenon F., Costero A.M., Gil S., Parra M. (2015). Azide and sulfonylazide functionalized fluorophores for the selective and sensitive detection of hydrogen sulfide. Sens. Act. B Chem..

[24] Wang X., An L., Tian Q., Cui K. (2019). Recent progress in H 2 S activated diagnosis and treatment agents. RSC Adv..

[25] Liu J., Liu X., Lu S., Zhang L., Feng L., Zhong S., Zhang N., Bing T., Shangguan D. (2020). Ratiometric detection and imaging of hydrogen sulfide in mitochondria based on a cyanine/naphthalimide hybrid fluorescent probe. Analyst.

[26] Henthorn H.A., Pluth M.D. (2015). Mechanistic insights into the H_2_S-mediated reduction of aryl azides commonly used in H_2_S detection. J. Am. Chem. Soc..

[27] Xu Q., He L., Wei H., Lin W. (2018). An ICT-based hydrogen sulfide sensor with good water solubility for fluorescence imaging in living cells. J. Fluoresc..

[28] Mowbray S.E., Amiri A.M. (2019). A brief overview of medical fiber optic biosensors and techniques in the modification for enhanced sensing ability. Diagnostics.

[29] Singh L., Agarwal N., Barthwal H., Arya B., Singh T. (2021). Application of Fiber Optics in Bio-Sensing. Fiber Optics-Technology and Applications.

[30] Li C., Xiao L., Yang X., Feng W. (2021). Ag/APTES/Cu_x_O (x = 1, 2)-MGS-coated no-core fibre surface plasmon resonance gas sensor and its application in hydrogen sulfide detection. IEEE Sens. J..

[31] Chen R., Lan G., Wang N., Yan W., Yi J., Wei W. (2021). Highly sensitive fibre-optic SPR sensor with surface coated TiO_2_/MWCNT composite film for hydrogen sulfide gas detection. J. Phys. D Appl. Phys..

[32] Lopez J.D., Keley M., Dante A., Werneck M.M. (2021). Optical fibre sensor coated with copper and iron oxide nanoparticles for hydrogen sulfide sensing. Opt. Fibre Technol..

[33] Chen R., Liu W., Huang G., Wang D., Qin X., Feng W. (2018). Hydrogen sulfide sensor based on tapered fibre sandwiched between two molybdenum disulfide/citric acid composite membrane coated long-period fibre gratings. Appl. Opt..

[34] Qin X., Feng W., Yang X., Wei J., Huang G. (2018). Molybdenum sulfide/citric acid composite membrane-coated long period fibre grating sensor for measuring trace hydrogen sulfide gas. Sens. Act. B Chem..

[35] Feng X., Feng W., Tao C., Deng D., Qin X., Chen R. (2017). Hydrogen sulfide gas sensor based on graphene-coated tapered photonic crystal fibre interferometer. Sens. Act. B Chem..

[36] Huang G., Li Y., Chen C., Yue Z., Zhai W., Li M., Yang B. (2020). Hydrogen sulfide gas sensor based on titanium dioxide/amino-functionalized graphene quantum dots coated photonic crystal fibre. J. Phys. D Appl. Phys..

[37] De Acha N., Socorro-Leránoz A.B., Elosúa C., Matías I.R. (2021). Trends in the design of intensity-based optical fiber biosensors (2010–2020). Biosensors.

[38] Li Y., Luo S., Gui Y., Wang X., Tian Z., Yu H. (2023). Difunctional hydrogel optical fiber fluorescence sensor for continuous and simultaneous monitoring of glucose and pH. Biosensors.

[39] Gong J., Tanner M.G., Venkateswaran S., Stone J.M., Zhang Y., Bradley M. (2020). A hydrogel-based optical fibre fluorescent pH sensor for observing lung tumor tissue acidity. Anal. Chim. Acta.

[40] Nguyen T.H., Sun T., Grattan K.T. (2019). A turn-on fluorescence-based fibre optic sensor for the detection of mercury. Sensors.

[41] Sarkar P.K., Halder A., Adhikari A., Polley N., Darbar S., Lemmens P., Pal S.K. (2018). DNA-based fiber optic sensor for direct in-vivo measurement of oxidative stress. Sens. Act. B Chem..

[42] Epstein J.R., Walt D.R. (2003). Fluorescence-based fibre optic arrays: A universal platform for sensing. Chem. Soc. Rev..

[43] Smith S., Goodge K., Delaney M., Struzyk A., Tansey N., Frey M. (2020). A comprehensive review of the covalent immobilization of biomolecules onto electrospun nanofibers. Nanomaterials.

[44] Kandimalla V.B., Tripathi V.S., Ju H. (2008). Biosensors based on immobilization of biomolecules in sol-gel matrices. Electrochemical Sensors, Biosensors and their Biomedical Applications.

[45] Hao G., Xu Z.P., Li L. (2018). Manipulating extracellular tumour pH: An effective target for cancer therapy. RSC Adv..

[46] Lee S., Shanti A. (2021). Effect of exogenous ph on cell growth of breast cancer cells. Int. J. Mol. Sci..

[47] Gottlieb H.E., Kotlyar V., Nudelman A. (1997). NMR chemical shifts of common laboratory solvents as trace impurities. J. Org. Chem..

[48] Betancourt F., Valente A., Yan H. (2021). 1, 8-Naphthalimide derivatives as probes for protein surface hydrophobicity. J. Photochem. Photobiol. A Chem..

[49] Montoya L.A., Pluth M.D. (2012). Selective turn-on fluorescent probes for imaging hydrogen sulfide in living cells. Chem. Commun..

[50] Rouhani S., Haghgoo S. (2015). A novel fluorescence nanosensor based on 1, 8-naphthalimide-thiophene doped silica nanoparticles, and its application to the determination of methamphetamine. Sens. Act. B Chem..

[51] Mousli Y., Rouvière L., Traboulsi I., Hunel J., Buffeteau T., Heuzé K., Vellutini L., Genin E. (2018). Hydrosilylation of azide-containing olefins as a convenient access to azidoorganotrialkoxysilanes for self-assembled monolayer elaboration onto silica by spin coating. Chemistryselect.

[52] Multar E., Daud S., Rohizad S.N.A., Halid N.T. (2020). Functionalized fibre optics for glucose detection. J. Phys. Conf. Ser..

[53] Tosi D., Sypabekova M., Bekmurzayeva A., Molardi C., Dukenbayev K. (2021). Optical Fibre Biosensors: Device Platforms, Biorecognition, Applications.

[54] Sypabekova M., Hagemann A., Rho D., Kim S. (2022). 3-Aminopropyltriethoxysilane (APTES) deposition methods on oxide surfaces in solution and vapor phases for biosensing applications. Biosensors.

[55] Zhu M., Lerum M.Z., Chen W. (2012). How to prepare reproducible, homogeneous, and hydrolytically stable aminosilane-derived layers on silica. Langmuir.

[56] Pasternack R.M., Rivillon Amy S., Chabal Y.J. (2008). Attachment of 3-(aminopropyl) triethoxysilane on silicon oxide surfaces: Dependence on solution temperature. Langmuir.

[57] Chastain J., King R.C. (1992). Handbook of X-ray Photoelectron Spectroscopy.

[58] Major G.H., Fernandez V., Fairley N., Smith E.F., Linford M.R. (2022). Guide to XPS data analysis: Applying appropriate constraints to synthetic peaks in XPS peak fitting. J. Vac. Sci. Technol. A Vacuum, Surfaces, Film..

[59] Dalby K.N., Nesbitt H.W., Zakaznova-Herzog V.P., King P.L. (2007). Resolution of bridging oxygen signals from O 1s spectra of silicate glasses using XPS: Implications for O and Si speciation. Geochim. Cosmochim. Acta.

[60] Miller D.J., Biesinger M.C., McIntyre N.S. (2002). Interactions of CO_2_ and CO at fractional atmosphere pressures with iron and iron oxide surfaces: One possible mechanism for surface contamination?. Surf. Interface Anal..

[61] Min H., Girard-Lauriault P.L., Gross T., Lippitz A., Dietrich P., Unger W.E. (2012). Ambient-ageing processes in amine self-assembled monolayers on microarray slides as studied by ToF-SIMS with principal component analysis, XPS, and NEXAFS spectroscopy. Anal. Bioanal. Chem..

[62] Brandner S., Kratky T., Holtz K., Becker T., Jekle M. (2021). Controlling glass bead surface functionality-Impact on network formation in natural edible polymer systems. Compos. Sci. Technol..

[63] Barral K., Moorhouse A.D., Moses J.E. (2007). Efficient conversion of aromatic amines into azides: A one-pot synthesis of triazole linkages. Org. Lett..

[64] Jothi D., Munusamy S., Iyer S.K. (2021). A new sensitive “turn-on” fluorescent probe based on naphthalimide: Application in visual recognition of hydrogen sulfide in environmental samples and living cells. J. Photochem. Photobiol. Chem..

[65] Zhang Y., Zhang L. (2020). A novel “turn-on” fluorescent probe based on hydroxy functionalized naphthalimide as a logic platform for visual recognition of H_2_S in environment and living cells. Spectrochim. Acta Part A Mol. Biomol. Spectrosc..

