# Supplementary material for: Surface Functionalised Optical Fibre for Detection of Hydrogen Sulphide
Source: Biosensors (Basel). 2023 Oct 24;13(11):949. doi: 10.3390/bios13110949 (PMC10669384; doi:10.3390/bios13110949)
Supplement: Supplementary file 1 [file biosensors-13-00949-s001.zip › biosensors-2658438-Supplementary Materials.pdf]

Article

Surface Functionalised Optical Fibre for Detection of Hydrogen Sulphide

Shaghayegh Baghapour [1,*], Jasmine Nehema [2], Wen Qi Zhang [1,3], Stephen C. Warren-Smith [1,3], Shane M. Hickey [2], Sally E. Plush [2] and Shahraam Afshar Vahid [1]

1 Laser Physics and Photonic Devices Laboratory, UniSA STEM, University of South Australia, Adelaide, SA 5095, Australia
2 Clinical and Health Sciences, University of South Australia, Adelaide, SA 5000, Australia
3 Future Industries Institute, University of South Australia, Adelaide, SA 5095, Australia
* Correspondence: shaghayegh.baghapour@mymail.unisa.edu.au

^{1}H NMR and ^{13}C NMR of Compounds

Figures S1 to S8 represent ^{1}H NMR and ^{13}C NMR data of compounds **1**, **2**, **4** and **5**.

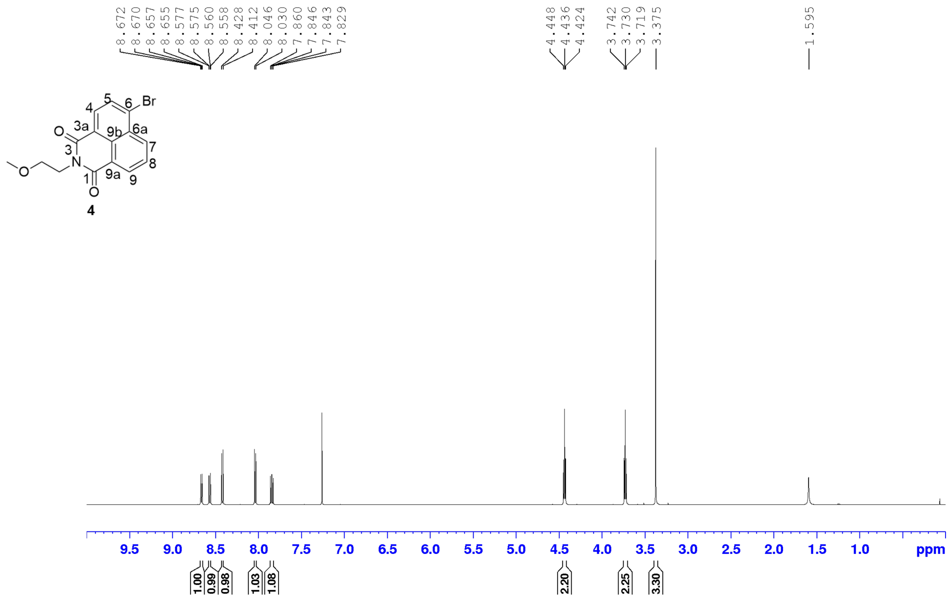

Figure S1. ^{1}H NMR (500 MHz) spectra of **4** in $CDCl_3$.

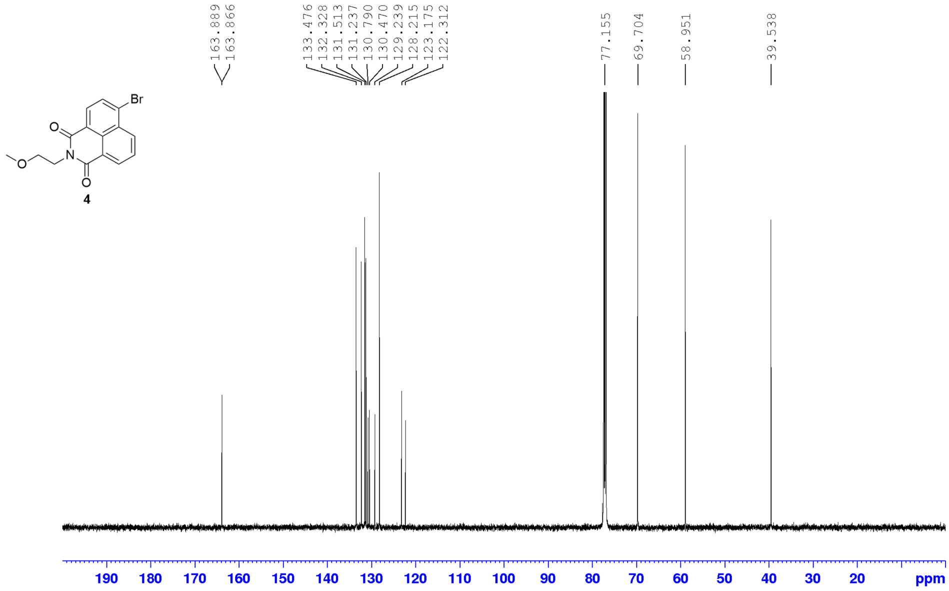

Figure S2. ^{13}C NMR (125 MHz) spectra of **4** in $CDCl_3$.

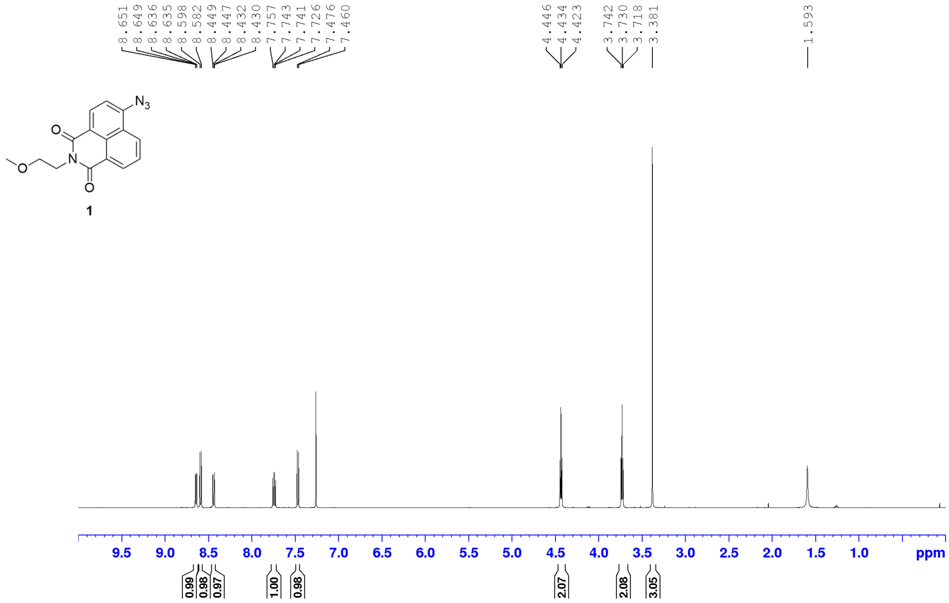

Figure S3. ^{1}H NMR (500 MHz) spectra of **1** in in $CDCl_3$.

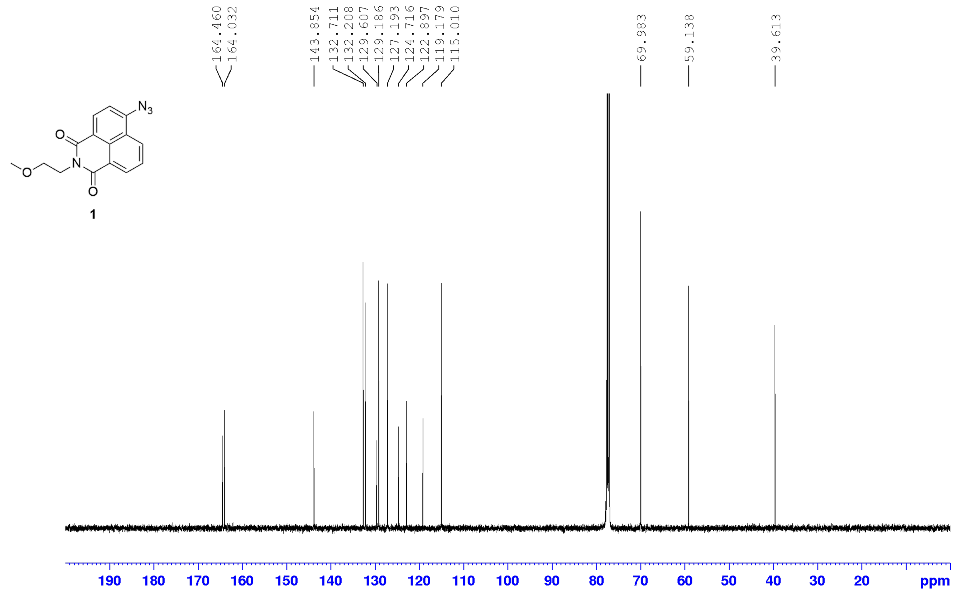

Figure S4. ^{13}C NMR (125 MHz) spectra of **1** in in $CDCl_3$.

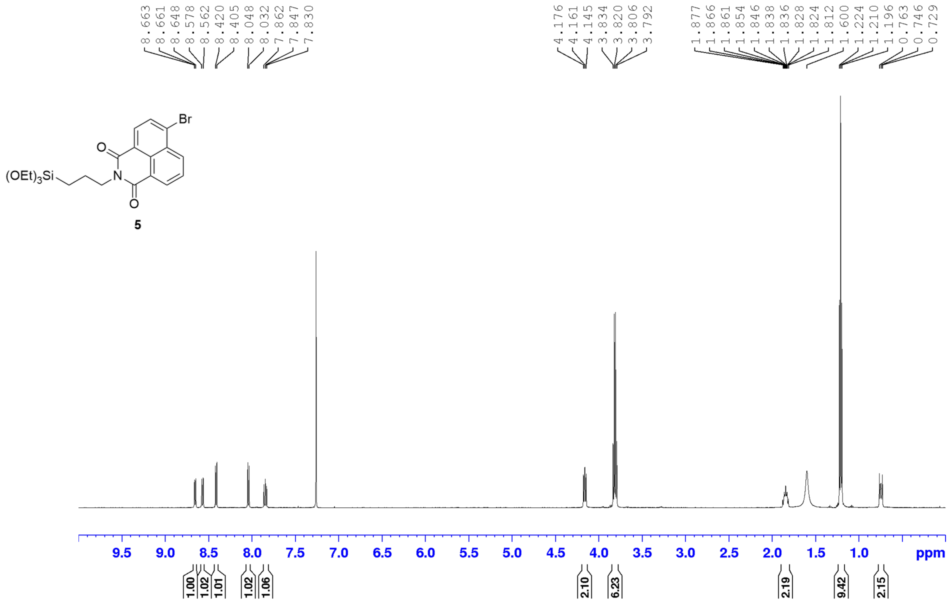

Figure S5. ^{1}H NMR (500 MHz) spectra of **5** in in $CDCl_3$.

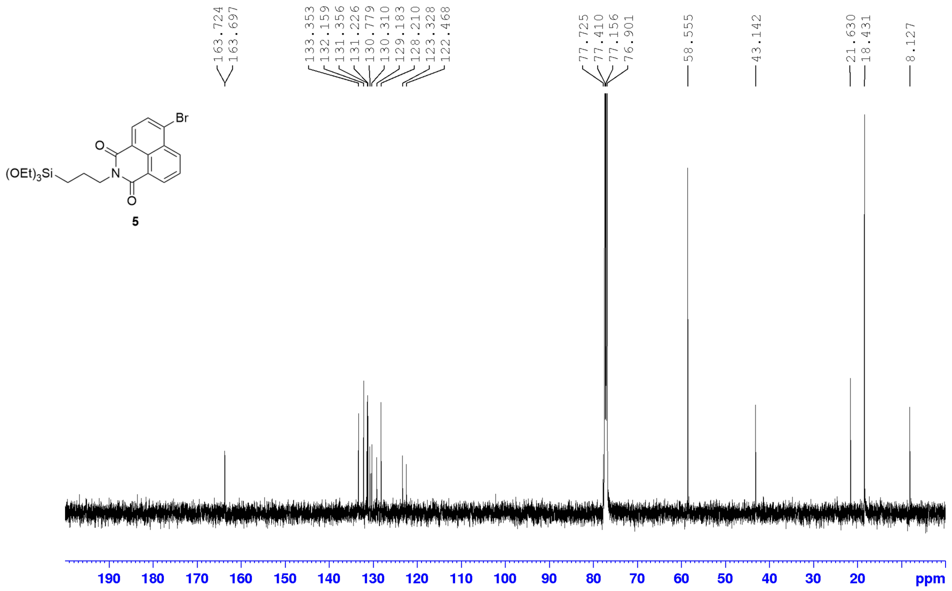

Figure S6. ^{13}C NMR (125 MHz) spectra of **5** in $CDCl_3$.

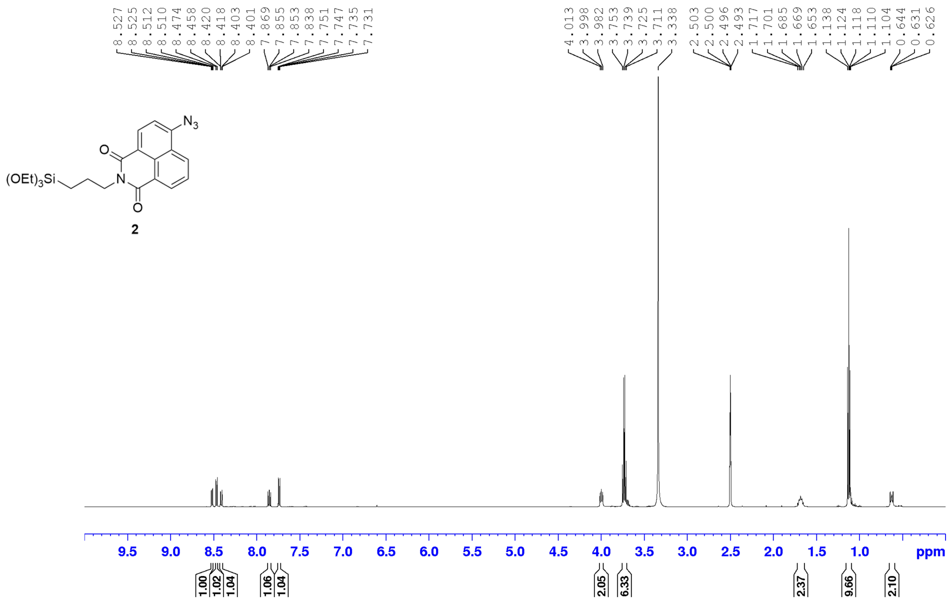

Figure S7. ^{1}H NMR (500 MHz) spectra of **2** in DMSO-*d6*.

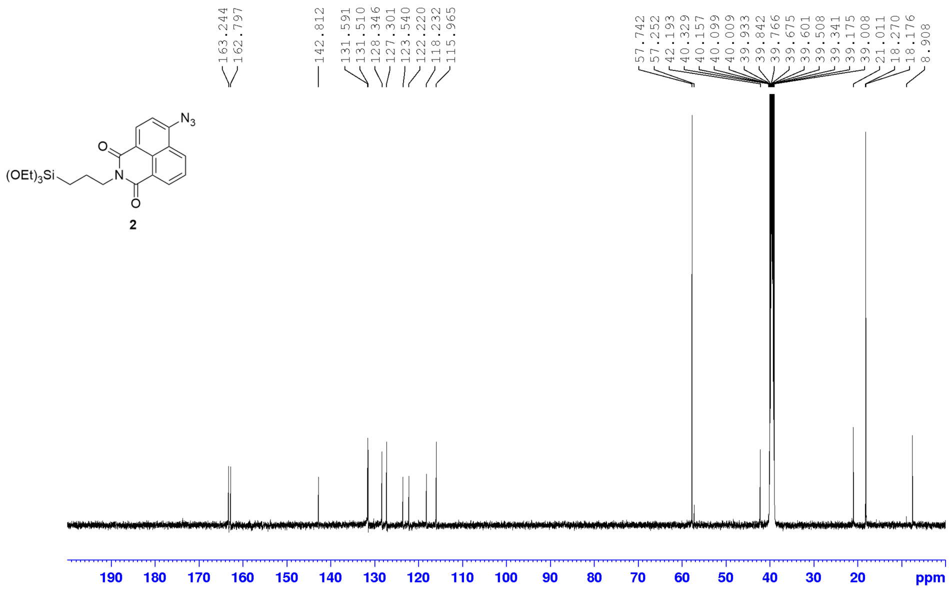

Figure S8. ^{13}C NMR (125MHz) spectra of **2** in in DMSO-*d6*.

Coating setup

Figure S9 shows the coating solution tube with optical fibres inside. A septa with needle was used to cap the tube. The tube was placed on a heater for 70 °C.

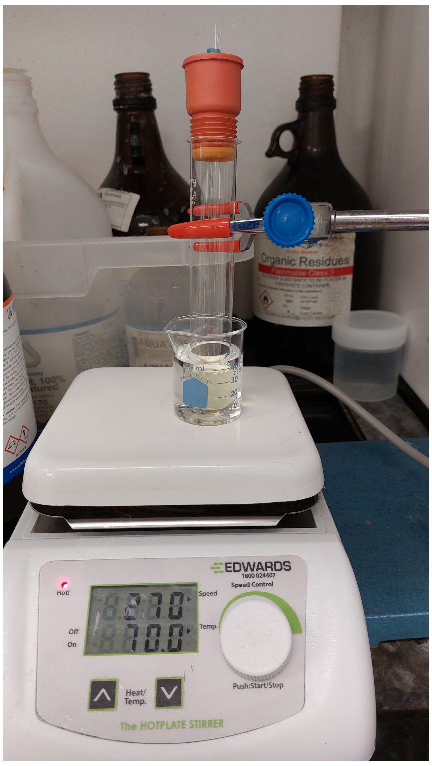

Figure S9. Coating setup. A glass tube containing the coating solution with optical fibres.

HR XP Spectra of C 1s, O 1s, Si 2p and N 1s of Control and Coated Fibres

Figure S10 presents the high-resolution spectra of C 1s, O 1s, Si 2p, and N 1s for control optical fibers.

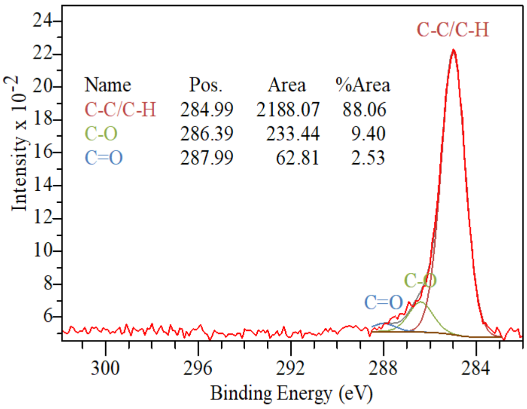

(a) HR C 1s of control fibres

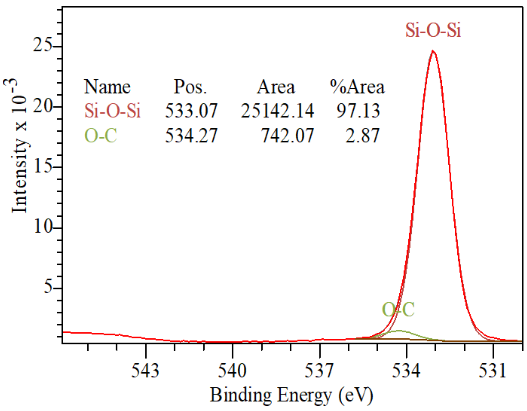

(b) HR O 1s of control fibres

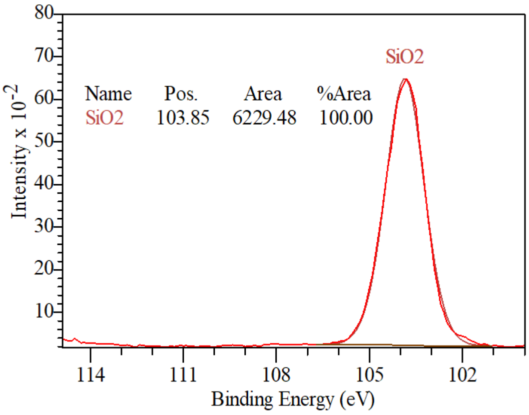

(c) HR Si 2p of control fibres

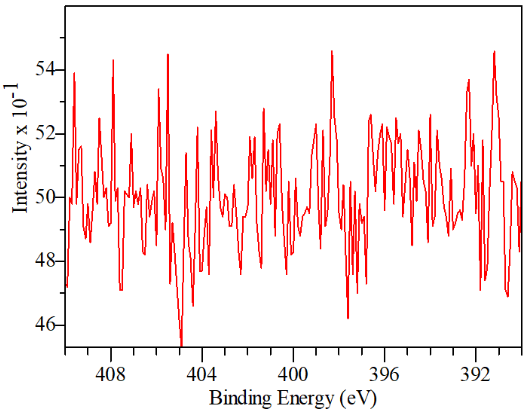

(d) HR N 1s of control fibres

Figure S10. High resolution spectra of C 1s, O 1s, Si 2p and N 1s of control fibres.

Figure S11 presents the high-resolution spectra of C 1s, O 1s, Si 2p, and N 1s for coated optical fibers.

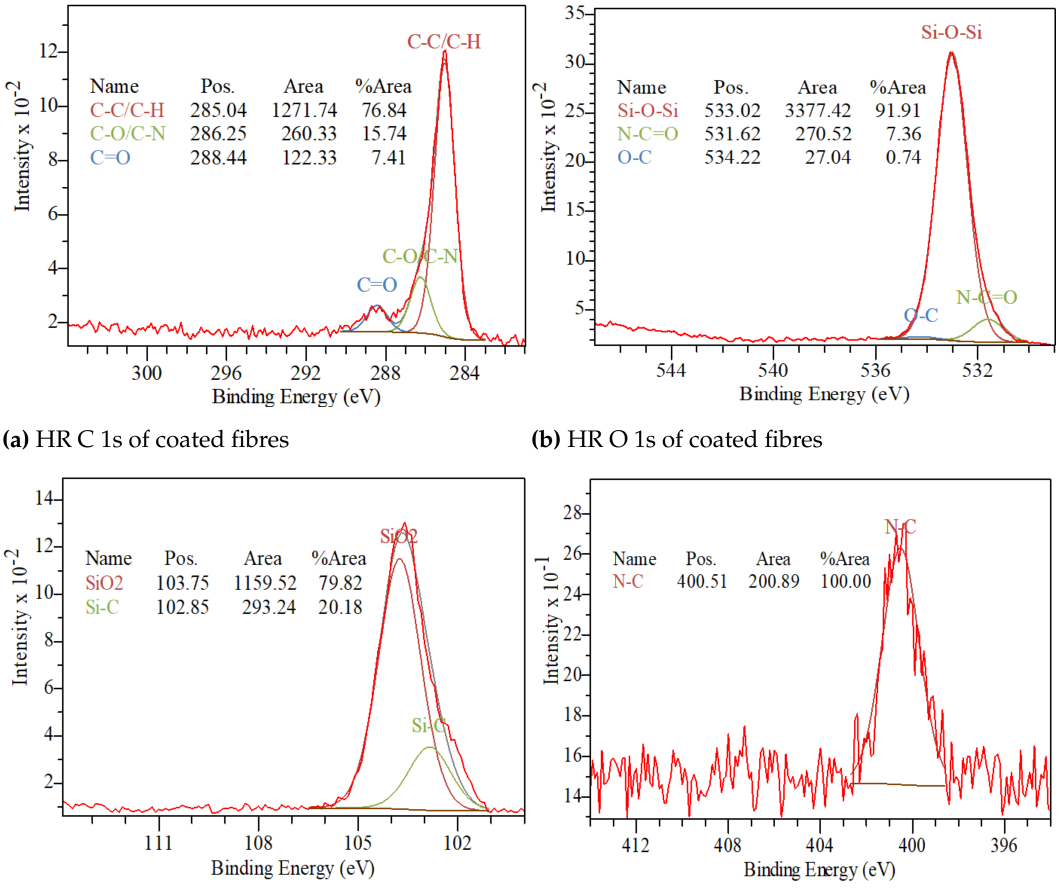

(a) HR C 1s of coated fibres

(b) HR O 1s of coated fibres

(c) HR Si 2p of coated fibres

(d) HR N 1s of coated fibres

Figure S11. High resolution spectra of C 1s, O 1s, Si 2p and N 1s of coated fibres.
